# Oral Intake of Linseed Oil Inhibits Skin Barrier Dysfunction in Obese Mice

**DOI:** 10.7759/cureus.61392

**Published:** 2024-05-30

**Authors:** Yoshiko Horie, Akiko Harauma, Toru Moriguchi, Hideaki Mitsui, Tomoko Akase

**Affiliations:** 1 Department of Biological Science and Nursing, Yokohama City University Graduate School of Medicine, Yokohama, JPN; 2 School of Life and Environmental Science, Azabu University, Sagamihara, JPN

**Keywords:** anti-inflammation, omega-3 fatty acids, linseed oil, skin barrier, obesity

## Abstract

Objective: Obesity is not only a risk factor for lifestyle-related diseases but also causes skin barrier dysfunction, which leads to a reduced quality of life due to dryness, itching, and scratching, and thus requires appropriate treatment. However, there are no studies on this issue. Therefore, this study aimed to examine whether oral intake of linseed oil is effective for skin barrier function in obesity and to confirm how the effect is demonstrated.

Methods: TSOD mice received either sterile distilled water (Control group) or linseed oil (Omega group), containing a high level of omega-3 fatty acids, including α-linolenic acid, orally for eight weeks. Mice were then irradiated with ultraviolet B (UVB) and three days later, transepidermal water loss (TEWL), which is the primary outcome of skin barrier function, was measured and gross skin appearance was observed. Hematoxylin and eosin (HE) staining and Ki-67 immunostaining were performed on skin samples. mRNA expression levels of the inflammatory markers *Tnfα*, *Cox2*, *Mcp1*, and *Hmox1* were measured by real-time reverse transcriptase-polymerase chain reaction (RT-PCR). We also performed fatty acid analysis of skin and erythrocytes by gas chromatography. Statistical analysis was performed using unpaired Student’s t-test and Pearson's correlation analysis.

Results: Compared with the Control group, the Omega group exhibited lower TEWL values and little skin erythema. Histological analysis revealed thinner epidermis and fewer Ki-67 positive cells. Additionally, in the Omega group, mRNA levels of four inflammation-related genes were lower, α-linolenic acid levels in both skin and erythrocytes were higher, and a lower n-6/n-3 ratio was observed. And α-linolenic acid levels in the skin were negatively correlated with the expression levels of inflammation-related genes.

Conclusion: Oral intake of linseed oil was found to inhibit skin barrier dysfunction in obesity. This effect was mediated by α-linolenic acid, a major component of linseed oil with anti-inflammatory properties, which was taken up by erythrocytes and supplied to the skin. Therefore, oral intake of linseed oil is expected to be a useful therapeutic method for skin barrier dysfunction in obesity.

## Introduction

In recent years, the number of obese people has been increasing worldwide [1]. Obesity is not only a risk factor for lifestyle-related diseases but also affects the skin, causing various skin problems such as bedsores and delayed wound healing [2]. Therefore, obesity-related skin problems are likely to increase in the future as the number of obese people increases.

In addition to the skin problems mentioned above, obese individuals have a higher incidence of atopic dermatitis and psoriasis, which are characterized by reduced skin barrier function [3]. In addition, reduced skin barrier function has also been reported in obese individuals without skin diseases [4-6]. Reduced skin barrier function leads to dryness, itching, and scratching, which in turn leads to mental and physical stress, poor body image, and other quality-of-life issues, highlighting the need for appropriate treatments. However, to date, no studies have explored treatment options for skin barrier dysfunction in obesity.

The skin barrier, primarily the epidermis, prevents excessive water evaporation from the body and keeps external substances from entering the body. Lipids in the epidermis help maintain skin barrier function and skin lipid composition is strongly influenced by dietary lipid intake. For example, skin barrier function is impaired in animals fed an essential fatty acid-deficient diet or a high-fat diet [7,8]. In addition, omega-3 fatty acid consumption promotes skin barrier function by increasing the omega-3 fatty acid content of the skin [9] and has been reported to improve skin barrier function in patients with atopic dermatitis and psoriasis [10,11]. These findings indicate that the types of lipids that are consumed have a strong influence on skin barrier function. However, in parallel with the recent increase in obesity, the intake of omega-3 fatty acids, which cannot be synthesized by the human body and therefore must be obtained through the diet, is currently declining [12]. Omega-3 fatty acids include α-linolenic acid, which is mainly derived from plants, as well as eicosatetraenoic acid (EPA) and docosahexaenoic acid (DHA), which are derived from fish. α-linolenic acid is abundant in linseed oil and is reported to have anti-inflammatory and antiallergic properties [13,14].

Many studies have reported that skin barrier dysfunction and inflammation often occur simultaneously [15,16]. In addition, obese skin, which is characterized by impaired barrier function, is more prone to inflammation than that of non-obesity, as ultraviolet B (UVB) irradiation increases the production of tumor necrosis factor-alpha (TNF-α), an inflammatory cytokine [17]. Furthermore, impaired skin barrier function caused by medication use in obese individuals results in increased transepidermal water loss (TEWL), which is a general measure method of skin barrier function, as compared with non-obese individuals [18]. Therefore, because the skin of obese individuals is characterized by high sensitivity to external stimuli and inflammation, and weight loss alone does not improve skin barrier function [19], there is a need for preventive care to address impaired skin barrier function in this population.

Thus, with the final goal of establishing a treatment for skin barrier dysfunction in obesity, this study aimed to examine whether oral intake of linseed oil is effective for skin barrier function in obesity and to confirm how the effect is demonstrated. The omega-3 fatty acid source used in this study was linseed oil, which was considered feasible because a small spoonful provides the recommended daily intake of omega-3 fatty acids. The effects of omega-3 fatty acids on skin barrier function in obesity have not been studied previously. Therefore, in this study, we performed experiments to generate basic findings that could be applied clinically.

## Materials and methods

This study was approved by the Yokohama City University Animal Experiment Committee (F-A-23-051), and was conducted in strict accordance with the basic guidelines for conducting animal experiments at research institutes (published by the Ministry of Education, Culture, Sports, Science and Technology).

Experimental animals and dietary intervention

To minimize the effect of blood glucose levels on skin barrier function, TSOD mice, a spontaneously obese mouse model that exhibits only a slow rise in blood glucose levels, were used. A total of four-week-old male TSOD mice were purchased from the General Research Institute of Animal Breeding (Ibaraki, Japan) and acclimated for one week. They were kept in a facility with a constant room temperature of 23±1°C, humidity of 55±5%, and 12 hours of light per day (7:00 am to 7:00 pm).

The TSOD mice were divided into two groups: the Control group (n=6) and the Omega group (n=6). No differences in body weight, blood glucose levels, and TEWL were observed between the groups. Non-obese TSNO mice, which are the control mice for TSOD mice and do not exhibit obesity, were used as standards for body weight and histological analysis. The Control group received sterile distilled water, and the Omega group received 0.1 ml/10 g/day linseed oil (Nisshin Oillio, Tokyo, Japan), containing omega-3 fatty acids, orally for eight weeks. Food and drinking water were provided ad libitum. The diet was solid diet MF (Oriental Yeast Co., Ltd., Tokyo, Japan); the composition of the solid diet MF is shown in Table 1, and the fatty acid composition of solid diet MF and linseed oil is shown in Table 2. Body weight and food intake were measured once a week.

**Table 1:** MF diet formulations (g per 100g of diet) Source: Oriental Yeast Co., Ltd. (https://www.oyc.co.jp/bio/LAD-equipment/LAD/ingredient.html)

Component	Content (g)
Water	8.1
Protein	23.2
Lipid	4.9
Crude ash	5.9
Coarse fiber	3.3
Nitrogen free extract	54.7
Energy (kcal/100g)	355.7

**Table 2:** Fatty acids composition of the MF diet and linseed oil (% of total fatty acids) Note: unpaired Student’s t-test was conducted, **p<0.01. N.D., not detected; Sat., saturated fatty acids; Mono., mono-unsaturated fatty acids; 18:3n3, α-linolenic acid; 20:5n3, eicosapentaenoic acid (EPA); 22:5n3, docosapentaenoic acid (DPA); 22:6n3, docosahexaenoic acid (DHA)

Fatty acids	MF diet, mean ± SE			Linseed oil, mean ± SE		
Sat.	20.128 ± 0.043			10.331 ± 0.047 **		
Mono.	26.009 ± 0.032			20.395 ± 0.016 **		
n-6	42.772 ± 0.056			15.134 ± 0.021 **		
n-3	6.450 ± 0.017			52.676 ± 0.081 **		
18:3n3	3.143 ± 0.005			52.619 ± 0.084 **		
20:5n3	1.576 ± 0.007			N.D.		
22:5n3	0.253 ± 0.004			N.D.		
22:6n3	1.478 ± 0.006			N.D.		
n-6/n-3 ratio	6.632 ± 0.023			0.287 ± 0.000 **		

Evaluation of skin barrier function

The primary outcome was changes in TEWL. After eight weeks of the intervention, the hair was removed from each mouse’s back, and UVB irradiation (40 mJ/cm^2^) was applied to the back skin. Three days after irradiation, when the TEWL value peaked, TEWL was measured using a Tewameter® instrument (Courage+Khazaka Electronic GmbH, Köln, Germany), and the gross appearance of the skin was observed (the gross appearance was confirmed by several researchers). Then, blood was drawn from the tail vein, and blood glucose levels were measured using an Accu-Chek instrument (Roche Holding AG, Basel, Switzerland). Next, the mice were anesthetized by isoflurane inhalation (1.5 mL/min), and blood was drawn from the inferior vena cava. Finally, the animals were euthanized by isoflurane inhalation (5 mL/min), and back skin samples were collected.

Histological analysis

A portion of the collected skin tissue was fixed in Mildform 10N (Wako Pure Chemical Industries, Osaka, Japan) overnight, paraffin-embedded, and thinly sectioned into 5-μm-thick slices. The slices were then deparaffinized, stained with hematoxylin and eosin (HE), and observed under an optical microscope (Olympus Corporation, Tokyo, Japan). The epidermal thickness was then quantified by examining the HE-stained skin tissue using ImageJ version 1.54 (National Institutes of Health, Maryland, United States). Three randomly selected images were taken per sample, and 10 locations were measured per image to calculate the average value for each group.

For Ki-67 immunostaining, deparaffinized slices prepared as described above were pretreated by autoclaved to induce antigen retrieval (121°C, 20 minutes) in citrate buffer (pH 7.6) (Wako Pure Chemical Industries). The sections were then immersed in methanol containing 3.0% hydrogen peroxide for 15 minutes to block endogenous peroxidase. Next, the sections were incubated with primary antibody diluted 1:150 (anti-Ki-67 rabbit monoclonal antibody (SP6)) (Abcam Limited, Cambridge, United Kingdom) for 60 minutes at room temperature. The sections were then incubated with secondary antibody (simple stain MAX-PO (MULTI)) (Nichirei Bioscience Corporation, Tokyo, Japan) for 30 minutes at room temperature, followed by chromogenic staining with DAB (Dako Denmark A/S, Glostrup, Denmark). The number of Ki-67 positive cells in three hot-spot images per sample was determined, and the average value for each group was calculated.

Real-time reverse transcriptase-polymerase chain reaction (RT-PCR)

Total RNA was isolated from a portion of the remaining skin tissue using the RNeasy Lipid Tissue Mini Kit (Qiagen, Hilden, Germany) according to the manufacturer’s instructions. The total RNA was reverse-transcribed into cDNA using a QuantiTect Reverse Transcription Kit (Qiagen). Gene expression levels were measured using a real-time PCR system (QuantStudio® 5 Real-Time PCR System; Thermo Fisher Scientific Inc., Waltham, Massachusetts, United States) and THUNDERBIRD® SYBR® qPCR MIX (Toyobo Co., Ltd., Osaka, Japan). The reaction conditions included 40 cycles of 20 seconds at 94°C, three seconds at 95°C, and 30 seconds at 60°C, and a melting curve analysis was performed. *Hprt1* was used as an internal standard. The expression levels of the target genes were determined by the ∆∆Ct method. The primer sequences used for real-time PCR amplification were as follows: *Tnf-α*, forward: 5′- ACCCTCACACTCAGATCATCTTC-3′, reverse: 5′-TGGTGGTTTGCTACGACGT-3′; *Cox-2*, forward: 5′-GCATTCTTTGCCCAGCACTT-3′; reverse: 5′-AGACCAGGCACCAGACCAAAGA-3′; *Mpc1*, forward: 5′-GCATCCACGTGTTGGCTCA-3′, reverse: 5′-CTCCAGCCTACTCATTGGGATCA-3′; *Hmox1*, forward: 5′-CCAGTGTATCGAGGGCCTTA-3′, reverse: 5′-CTGGTTGCCTTCATCCATCT-3′; *Hprt1*, forward: 5′-ATGCCCCAGGATTTGTCAGA-3′; reverse: 5′-GTTGCGCTCAATCTCCTCCT-3′.

Fatty acid analysis

Lipid Extraction and Methylation

The remaining skin tissue (approximately 25 mg) and erythrocytes (collected from the blood samples by centrifuging at 6000 rpm for 10 minutes) were frozen in liquid nitrogen immediately after collection and stored at -80°C until analysis. The samples were each placed in a solvent containing internal standard docosatrienoic acid methyl ester (22:3n-3) in 50 μg/ml butylated hydroxytoluene and 2 ml methanol:hexane (4:1) and homogenized under nitrogen gas. Then, 200 μl of acetyl chloride was added to each sample, the air in the tube was replaced with nitrogen, and the samples were heated at 100°C for one hour on a heating block. Next, the samples were rapidly cooled, 5 ml of 6% potassium carbonate solution was added to each sample, and the mixture was shaken to combine thoroughly. Finally, the mixture was centrifuged at 4000 rpm for two minutes, and the supernatant (containing the fatty acids) was collected and transferred to a microvial for gas chromatography. Lipids were extracted from the mouse chow and the linseed oil in the same manner.

Gas Chromatography

Fatty acid methyl esters were analyzed using a gas chromatograph (7890A; Agilent Technologies Ltd., California, United States) equipped with an automatic injector (7693A; Agilent Technologies Ltd) and detected using a hydrogen flame ionization detector. ChemStation software (Rev. B.04.01.SP1; Agilent Technologies Ltd) was used to control the instrument and collect data. DB-FFAP 15 m x 0.10 mm ID (Agilent Technologies Ltd) with 0.10-μm-thick film was used as the column for gas chromatography. Both the hydrogen flame ionization detector and injector temperatures were set at 250°C. The oven temperature was initially set to 150°C for 0.25 minutes, increased by 35°C/minute to 200°C, increased by 8°C/minute for 3.2 minutes until it reached 225°C, increased by 80°C/minute to 248°C, and then held at 248°C for 14.7 minutes. Hydrogen was used as the carrier gas, at a linear velocity of 56 cm/second. For accurate quantitation, a custom-mixed 28-component quantitative standard containing methyl esters with 10-24 carbon bonds and 0-6 double bonds was used to assign retention times (Prep 462; Nu-Chek Prep, Inc., Minnesota, United States). Fatty acid content is expressed as a percentage of the total peak area corresponding to weight percent (less than 5%). The concentration of fatty acids was calculated using internal standards. The limit of detection for fatty acid content was 0.01%. The mouse chow and the linseed oil were also performed in the same manner (Table 2).

Statistical analysis

Microsoft Excel version 16.82 (Microsoft Corporation, Redmond, Washington, United States) and IBM SPSS Statistics for Windows, Version 28.0 (Released 2021; IBM Corp., Armonk, New York, United States) were used for statistical analysis. Unpaired Student’s t-test was used for comparison between groups, and Pearson's correlation analysis was used to evaluate the correlation between α-linolenic acid levels in the skin and the mRNA expression of inflammation-related genes. All data are expressed as mean value ± standard error (SE). A *p*-value <0.05 was considered statistically significant.

## Results

Mouse body weight and blood glucose levels

**Figure 1:**
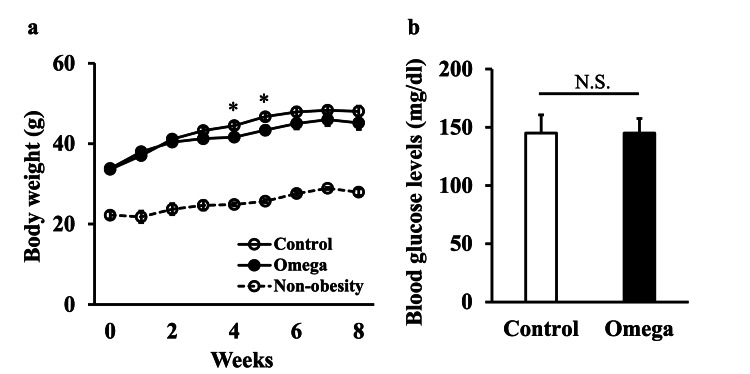
Body weight and blood glucose levels in the Control and Omega groups (a) Body weight was measured once a week throughout the eight-week experiment. TSNO mice were included as standard samples. (b) Blood glucose levels were measured at the end of the eight-week experiment. Values are shown as the mean ± SE (n=6). *p<0.05, N.S.: not significant, unpaired Student’s t-test

At four and five weeks, the Omega group exhibited lower body weights compared with the Control group. However, there was no significant difference in body weight between the two groups from six to eight weeks. The TSOD mice gained more weight than the TSNO mice at all time points (Figure 1a). Blood glucose levels were within normal limits for both the Control and Omega groups, with no statistically significant differences (Figure 1b). The average dietary intake per animal was 255.88 g/8 weeks in the Control group and 213.68 g/8 weeks in the Omega group. Of these, omega-3 fatty acid intake was 0.81 g/8 weeks in the Control group and 0.68 g/8 weeks in the Omega group. The difference in omega-3 fatty acid intake between the two groups was 0.133 g/8 weeks.

Skin barrier function and macroscopic skin findings

**Figure 2:**
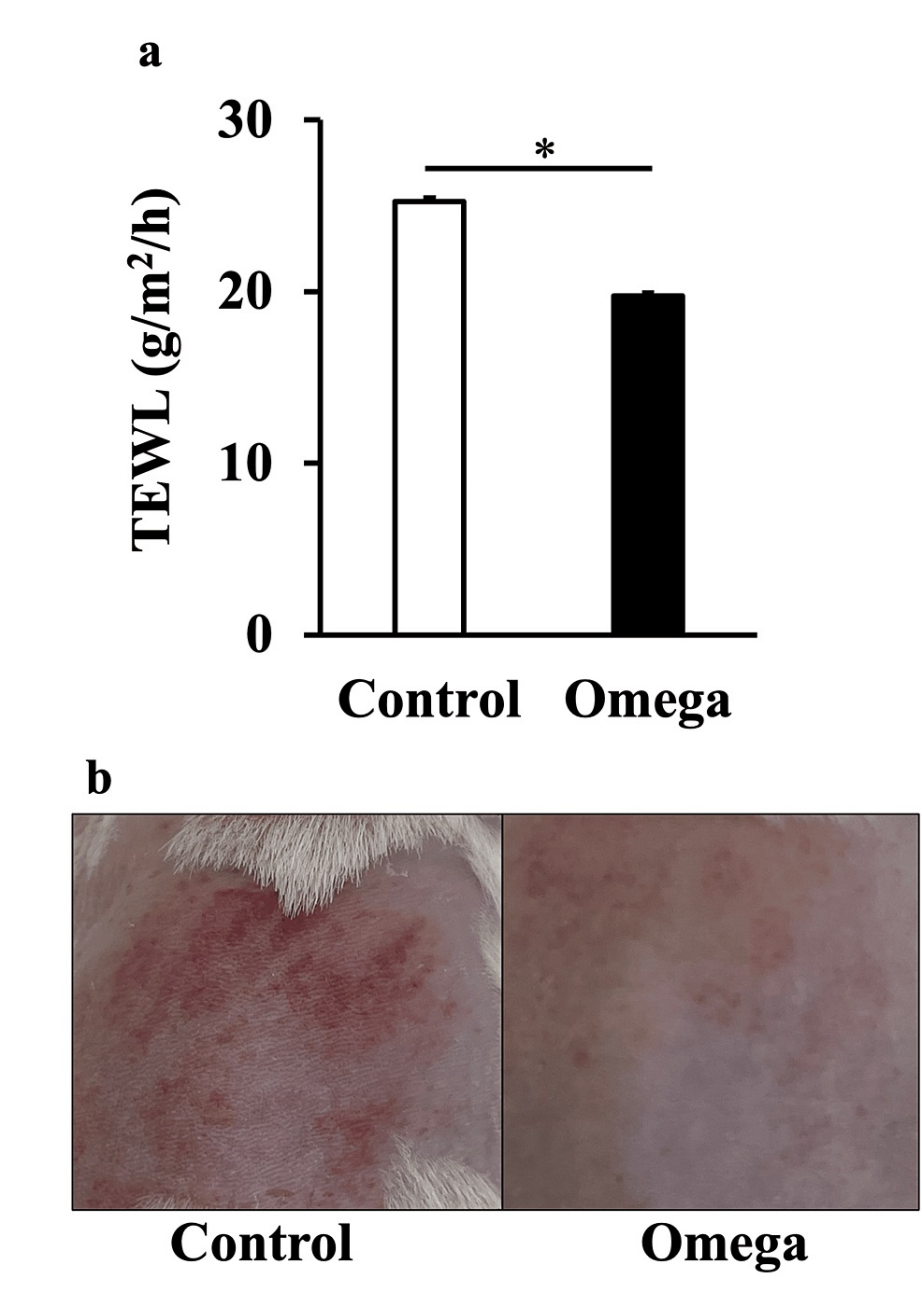
Effects of oral intake of linseed oil on skin barrier function in obese mice (a) TEWL and (b) gross appearance of the dorsal skin of TSOD mice three days after UVB irradiation. Values are shown as the mean ± SE (n=6). *p<0.05, unpaired Student’s t-test TEWL, transepidermal water loss; UVB, ultraviolet B

TEWL values were significantly lower in the Omega group than in the Control group (Figure 2a). Gross skin findings showed erythema in the Control group, but almost none in the Omega group (Figure 2b).

Histological analysis

**Figure 3:**
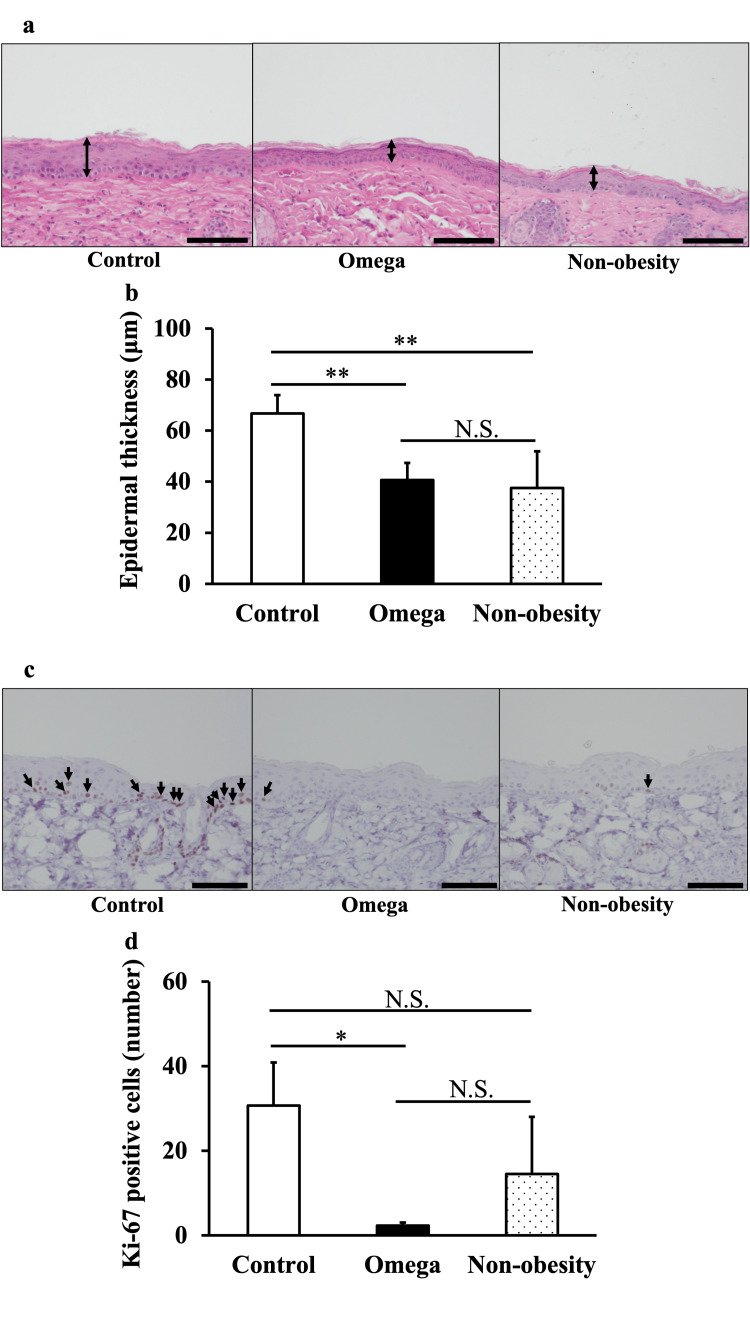
Histological analysis of skin from mice in the Control and Omega groups (a) The skin samples were stained with hematoxylin and eosin (HE) (scale bar = 100 µm). The arrows (↕︎) indicate epidermal thickness. (b) Epidermal thickness was measured. (c) Anti-Ki-67 staining of the skin samples (scale bar = 100 µm). Ki-67 positive cell (↓) indicated cell proliferation. (d) Quantification of Ki-67 positive cells in the different groups. TSNO mice were included as standard samples for the histological analysis. Values are shown as the mean ± SE (n=6); **p<0.01, *p<0.05, unpaired Student’s t-test

HE staining of skin tissue showed that the epidermis was thinner in the Omega group than in the Control group (Figure 3a), and quantification showed that this difference was significant (Figure 3b). The epidermal thickness in the Omega group was similar to that observed in non-obese mice (Non-obesity) (Figures 3a, 3b). Fewer Ki-67 positive cells were observed in the Omega group than in the Control group (Figure 3c), and this difference was significant (Figure 3d).

Real-time RT-PCR

**Figure 4:**
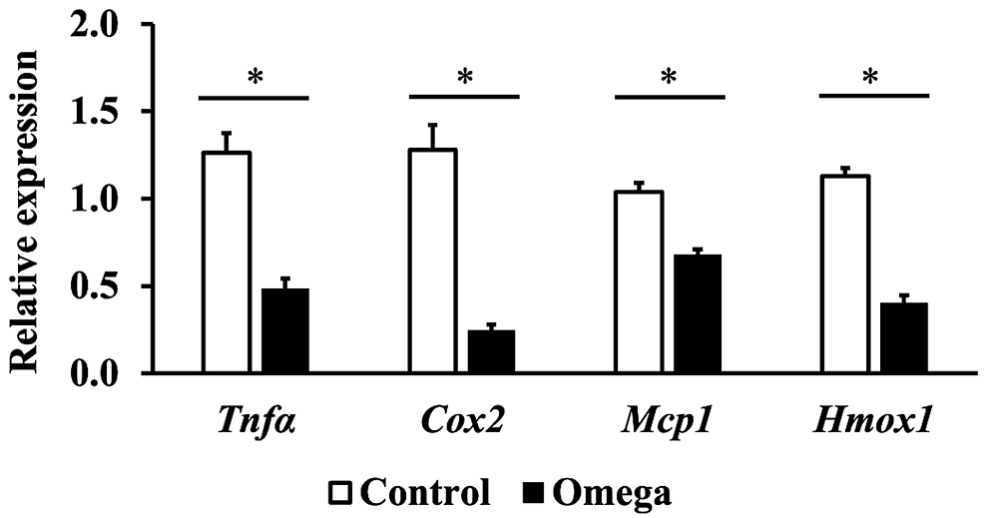
Relative mRNA expression levels of genes related to inflammation in the skin We measured the expression levels of *Tnfα*, *Cox2*, *Mcp1*, and *Hmox1*, inflammation-related genes whose expression is upregulated in obese skin three days after ultraviolet B irradiation. Values are shown as the mean ± SE (n=6). *p<0.05, unpaired Student’s t-test.

To investigate how oral intake of linseed oil inhibits skin barrier dysfunction in obese mice, we measured the expression levels of *Tnfα*, *Cox2*, *Mcp1*, and *Hmox1*, inflammation-related genes whose expression is upregulated in obese skin. The results showed that *Tnfα*, *Cox2*, *Mcp1*, and *Hmox1* expression levels were significantly decreased in the Omega group compared with those in the Control group (Figure 4).

Fatty acid content of skin and erythrocytes

To investigate how oral intake of linseed oil suppressed inflammation in the skin of obese mice, we performed a fatty acid analysis of the skin and erythrocytes. The results showed that the level of α-linolenic acid, an omega-3 fatty acid, was significantly increased in the skin of mice in the Omega group compared with that in the skin of mice in the Control group. A low n-6/n-3 ratio in the skin indicates better skin barrier function, and the n-6/n-3 ratio was significantly lower in the Omega group than in the Control group (Table 3). α-linolenic acid levels were also significantly increased in erythrocytes in the Omega group, and the n-6/n-3 ratio was significantly lower in erythrocytes in the Omega group (Table 4).

**Table 3:** Fatty acids composition of the skin (% of total fatty acids) Note: unpaired Student’s t-test was conducted, **p<0.01; (n=6) Sat., saturated fatty acids; Mono., mono-unsaturated fatty acids; 18:3n3, α-linolenic acid; 20:5n3, eicosapentaenoic acid (EPA); 22:5n3, docosapentaenoic acid (DPA); 22:6n3, docosahexaenoic acid (DHA)

Fatty acids	Control group, mean ± SE			Omega group, mean ± SE		
Sat.	22.732 ± 0.332			17.956 ± 0.133 **		
Mono.	43.284 ± 0.328			38.188 ± 1.067 **		
n-6	29.481 ± 0.281			27.125 ± 0.374 **		
n-3	1.659 ± 0.083			13.943 ± 0.818 **		
18:3n3	1.184 ± 0.024			13.266 ± 0.861 **		
20:5n3	0.097 ± 0.020			0.141 ± 0.007		
22:5n3	1.659 ± 0.083			0.120 ± 0.015		
22:6n3	0.297 ± 0.040			0.271 ± 0.065		
n-6/n-3 ratio	17.980 ± 0.851			1.980 ± 0.122 **		

**Table 4:** Fatty acids composition of the erythrocyte (% of total fatty acids) Note: unpaired Student’s t-test was conducted, **p<0.01; (n=5-6) Sat., saturated fatty acids; Mono., mono-unsaturated fatty acids; 18:3n3, α-linolenic acid; 20:5n3, eicosapentaenoic acid (EPA); 22:5n3, docosapentaenoic acid (DPA); 22:6n3, docosahexaenoic acid (DHA)

Fatty acids	Control group, mean ± SE			Omega group, mean ± SE		
Sat.	43.647 ± 0.121			43.938 ± 0.364		
Mono.	18.157 ± 0.323			16.667 ± 0.136 **		
n-6	26.248 ± 0.204			24.691 ± 0.339 **		
n-3	11.064 ± 0.178			13.587 ± 0.159 **		
18:3n3	0.127 ± 0.019			1.464 ± 0.069 **		
20:5n3	1.279 ± 0.038			2.705 ± 0.088		
22:5n3	1.218 ± 0.024			2.107 ± 0.055		
22:6n3	8.439 ± 0.151			7.126 ± 0.063		
n-6/n-3 ratio	2.375 ± 0.049			1.819 ± 0.041 **		

Correlation between α-linolenic acid levels in the skin and inflammation-related gene expression

To confirm that α-linolenic acid in the skin contributes to the mRNA expression level of inflammation-related genes, we evaluated the correlations between α-linolenic acid levels in the skin and mRNA expression of inflammation-related genes. As a result, α-linolenic acid levels were negatively correlated with the expression levels of inflammation-related genes (Table 5).

**Table 5:** Correlation between α-linolenic acid levels in the skin and inflammation-related gene expression Note: Pearson's correlation coefficient (r) was calculated, *p<0.05, #*p*≤0.05; (n=6)

Gene	r
Tnfα	-0.57^#^
Cox2	-0.65*
Mcp1	-0.61*
Hmox1	-0.69*

## Discussion

Our results indicate that oral intake of linseed oil for eight weeks inhibits the deterioration of skin barrier function in obese mice. Furthermore, we showed this effect was conferred by erythrocytes taking up α-linolenic acid, a major component of linseed oil with anti-inflammatory properties [13,20], and delivering it to the skin.

The TEWL, which was the primary outcome used to assess skin barrier function, and gross appearance of the skin indicated that oral linseed oil intake for eight weeks effectively inhibited skin barrier dysfunction in obese mice. In a clinical study of adult women with self-reported skin sensitivity, oral linseed oil supplementation for 6 or 12 weeks lowered TEWL levels, erythema, and blood flow to the skin; importantly, these effects correlated with plasma α-linolenic acid levels [21]. In rats that consumed EPA (which, like linseed oil, contains high levels of omega-3 fatty acids) for 30-90 days and were then treated with a drug to induce skin barrier dysfunction, skin barrier function was significantly greater than that seen in a control group pretreated with water, and the n-6/n-3 ratio was significantly lower [9]. Our study on obese skin similarly found that oral intake of linseed oil, which contains omega-3 fatty acids, inhibited skin barrier dysfunction, suggesting that α-linolenic acid supplied to the skin by erythrocytes inhibits inflammation. A previous study reported that hyperglycemia decreases skin barrier function [22]. In this study, blood glucose levels remained within normal limits in both groups, and there was no difference in blood glucose levels between the two groups (*p*=1.00), confirming that blood glucose had no effect on skin barrier function.

Histologically, impaired skin barrier function results in epidermal thickening and proliferation of Ki-67 positive cells [23,24]. In our study, we observed similar effects in the Control group but not in the Omega group. Furthermore, the histological findings from the Omega group were similar to those in non-obese mice, suggesting that oral intake of linseed oil promoted skin barrier function in obese mice. In a study that used a mouse model of contact dermatitis, dietary linseed oil intake suppressed epidermal thickening, and 12-hydroxyeicosapentaenoic acid (12-HEPE), a metabolite of linseed oil and EPA, suppressed the expression of genes encoding pre-inflammatory chemokines (*Cxcl1* and *Cxcl2*) via retinoid X receptors (*Rxrα*) expressed on keratinocytes in the epidermis, thereby reducing skin inflammation [14]. In the present study, we did not observe any changes in *Rxrα*, *Cxcl1*, or *Cxcl2* expression between skin samples from the Control and Omega groups (data not shown). Therefore, we hypothesize that linseed oil enhanced skin barrier function in obese mice by another factor. In addition, the epidermal thickening and proliferation of Ki-67 positive cells observed in the Control group are consistent with an earlier study, which showed increased *Tnfα* levels [16], as well as skin barrier dysfunction, in inflamed skin. Furthermore, skin inflammation in the context of obesity is also associated with increased *Tnfα* expression [25].

We, therefore, suspected that increased expression of inflammatory factors such as *Tnfα*, which is increased in the skin of obese individuals, was the factor responsible for the increased TEWL values and altered histological features observed in the Omega group. Thus, we measured the gene expression levels of inflammatory markers and found that *Tnfα*, *Cox2*, *Mcp1*, and *Hmox1* expression levels were significantly lower in the Omega group than in the Control group. In addition, fatty acid analysis showed that levels of α-linolenic acid, an omega-3 fatty acid, were significantly increased in the skin of the Omega group compared with the Control group. Furthermore, we confirmed that α-linolenic acid in the skin contributed to the inhibition of inflammation, as the expression of inflammation-related genes decreased with the increase of α-linolenic acid levels in the skin. Previous studies have reported that *Tnfα* and *Cox2* expression increase in UVB-exposed skin and that this effect can be suppressed by dietary supplementation with omega-3 fatty acids [26,27]. Omega-3 fatty acids have also been shown to decrease *Mcp1* and *Hmox1* expression levels in human keratinocytes and mouse fetal fibroblasts [28,29]. Thus, the improvement in skin barrier function in obese mice induced by oral linseed oil intake, as observed in the current study, could have been caused by increased omega-3 fatty acid content in the skin, which could suppress inflammation. Furthermore, previous studies have reported that increased omega-3 fatty acid levels in plasma, erythrocytes, and skin resulting from dietary omega-3 fatty acid intake are associated with improved skin barrier function and reduced skin inflammation [9,30]. This indicates that ingested omega-3 fatty acids are supplied to the skin through the bloodstream. Our findings suggest that, even in the context of obesity, ingested omega-3 fatty acids are supplied to the skin by erythrocytes, where they inhibit skin barrier dysfunction by exerting anti-inflammatory effects.

Limitations

Metabolites of omega-3 fatty acids were not measured in this study and we consider it necessary to measure the metabolites in the next stage of this study in order to explore the mechanism for the effect of oral intake of linseed oil on the inhibition of skin barrier function in obesity. Also, the duration of the intervention applied in this study was selected based on previous studies [9]. For clinical application, more research is needed to determine the optimal dose and intervention duration.

## Conclusions

This study showed that oral intake of linseed oil for eight weeks inhibited the deterioration of skin barrier function in obese mice. This effect was mediated by α-linolenic acid, a major component of linseed oil with anti-inflammatory properties, which was taken up by erythrocytes and supplied to the skin. Therefore, oral intake of linseed oil could be used to inhibit skin barrier dysfunction in obesity. In future studies, we investigate the optimal dose and duration of supplementation to apply the findings to a clinical context.
